# Non-photonic sensing of membrane-delimited reactive species with a Na^**+**^ channel protein containing selenocysteine

**DOI:** 10.1038/srep46003

**Published:** 2017-04-05

**Authors:** Navin K. Ojha, Enrico Leipold, Roland Schönherr, Toshinori Hoshi, Stefan H. Heinemann

**Affiliations:** 1 Center for Molecular Biomedicine, Department of Biophysics, Friedrich Schiller University Jena & Jena University Hospital, Jena, Germany; 2 Department of Physiology, University of Pennsylvania, Philadelphia, USA

## Abstract

Photonic experiments are of key importance in life sciences but light-induced side effects are serious confounding factors. Here we introduce roNa_V_2, an engineered voltage-gated Na^+^ channel harboring a selenocysteine in its inactivation motif, as a non-photonic, sensitive, gateable, and reversible sensor for membrane-delimited reactive species. roNa_V_2 allows for the assessment of chemical modification induced in fluorescence microscopy settings with high sensitivity and time resolution and it demonstrates the usefulness of ion channels as highly sensitive reporters of membrane processes.

Cellular reactive species (RS), e.g., superoxide (O_2_^−⋅^), hydroxide (HO^⋅^), hydrogen peroxide (H_2_O_2_), and hypochlorous acid (HClO) play crucial roles in physiological processes, such as signal transduction, activation of nuclear transcription factors, gene expression, and immune regulation^1^,^2^,^3^. However, excess RS modify lipids, proteins, and nucleic acids, and are often associated with adverse consequences such as neurodegeneration, atherosclerosis, diabetes, sepsis, and cancer^4^.

RS differ in their production, concentration, distribution, lifetime, molecular targets, and biological functions. Therefore, there is a clear demand for sensitive probes to precisely monitor cellular RS with spatio-temporal resolution to understand the multifaceted role of cellular RS in cell physiology. Recently, mutants and fusions of genetically encoded GFP (green fluorescent protein), for example, reduction and oxidation sensitive variants roGFP2^5^ and Grx1-roGFP2 (Grx = glutaredoxin)^6^, received much attention for cellular RS monitoring. roGFP2 is a genetically engineered fluorescent redox reporter, which was developed by inserting a dithiol-disulfide pair into GFP. The breakage and the formation of the disulfide bridge in roGFP2 leads to a differential change in fluorescence at 400 nm and 470 nm excitation wavelength, thus yielding a ratiometric signal that reports on oxidative changes in its local environment. Fusion of human glutaredoxin-1 to roGFP2 provides strongly accelerated formation of a disulfide bridge and, hence, increases the sensitivity of Grx1-roGFP2 in the physiologically relevant range for glutathione-mediated RS modifications^6^.

These fluorescent reporters offer several useful features, such as the choice of localized sub-cellular targeting, real-time RS detection, and ratiometric observation that overcome artifacts arising due to photo-bleaching and inhomogeneous distribution of the sensors. However, there is the inherent problem of phototoxicity that can lead to lasting irreversible destruction of cellular structures and may even result in immediate alteration of molecular function. Despite the obvious detrimental consequences of visible light in cell physiological studies, the issue of phototoxicity has been only infrequently considered in life science research^7^. The presence of intracellular flavin-containing oxidases, the cytochrome system, heme-containing proteins, and tryptophan-rich proteins not only confound with fluorescence techniques, they may also act as cellular photosensitizers and constitute the major source of light-induced RS^8^,^9^,^10^. Moreover, photo-activated GFP disturbs cellular redox homeostasis either by producing endogenous superoxide or altering the glutathione homeostasis^11^,^12^,^13^. Therefore, the potential interference of blue light as used for GFP-based imaging with the cellular redox system clearly demands for alternative tools or probes for monitoring cellular RS.

Previously, we have reported a genetically engineered voltage-gated Na^+^ (Na_V_) channel with a cysteine residue in its inactivation domain (roNa_V_1; rNa_V_1.4 mutant M1305C, also termed IFC) as a non-photonic RS sensor delimited to the cell membrane^14^. Na_V_ channels activate (open) and inactivate (close) rapidly in response to membrane depolarization, thus yielding transient Na^+^ inward current. Rapid and essentially complete inactivation of the Na_V_ channel, which is crucial for maintaining cellular excitability, is mediated by the inactivation motif “IFM” (Ile-Phe-Met), located in the intracellular linker between domains III and IV of the Na_V_ protein^15^. Variation of this motif, either by mutagenesis or by chemical modification, has a strong impact on inactivation and, hence, yields a ratiometric signal (ratio of non-inactivated current component and peak current) that can be measured with high precision. roNa_V_1 exhibits an RS sensitivity comparable to roGFP2, including a relatively slow response to changes in the cellular redox milieu^14^.

The speed of the roNa_V_1 response is limited by the kinetics by which the cysteine residue harbored in the inactivation motif of the channel is modified. We therefore thought to create a second-generation genetically engineered Na_V_ channel containing selenocysteine (Sec or U) in its inactivation motif, also referred as roNa_V_2. As outlined below, it may serve as a non-photonic tool for monitoring RS delimited to the cell membrane – in particular those generated by light exposure – in a switchable and ratiometric manner.

## Results and Discussion

### A Na_V_ channel based on a selenoprotein

**Figure 1:**
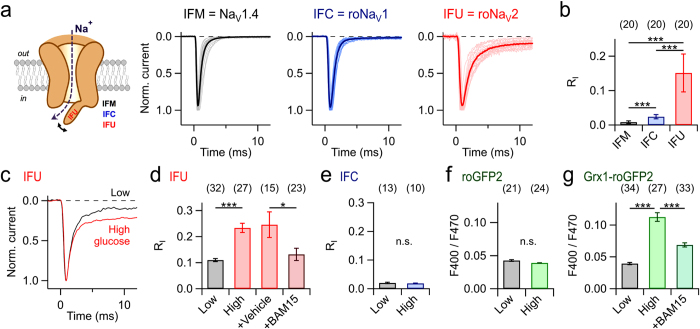
A sensor for membrane-delimited RS-mediated chemical modification. (**a**) Illustration of RS-sensitive Na_V_ channel (*left*) and current traces at −20 mV of the indicated Na_V_ channels expressed in HEK 293 cells (*right*). In each, 20 traces from different cells are superimposed (light); the thick traces indicate means. (**b**) Mean relative non-inactivated current 5 ms after depolarization to −20 mV; error bars indicate s.d. values. (**c**) Superposition of two sample current traces at −20 mV from cells expressing roNa_V_2, cultured in medium with low (5.5 mM) and high (25 mM) concentrations of glucose. (**d**) Mean residual current after 10 ms for roNa_V_2 for low (gray) and high glucose (red); “+Vehicle” refers to application of 0.1% DMSO, “+BAM15” to cells cultured in high-glucose medium in the presence of 5 μM of the mitochondrial uncoupler BAM15. (**e**) Mean residual current after 10 ms for roNa_V_1 in cells in low and high glucose conditions. (**f**) Mean fluorescence ratios (400 nm/470 nm) of roGFP2 expressed in cells cultured in low and high glucose media. (**g**) Mean fluorescence ratios (400 nm/470 nm) of Grx1-roGFP2 expressed in cells treated with the indicated glucose and BAM15 protocol as in (**d**). Data in (**d**–**g**) are mean ± s.e.m. with *n* indicated in parentheses; ***P < 0.001; *P < 0.05; n.s. *P* > 0.05 for a two-sided *t*-test.

By means of the hinged-lid mechanism, roNa_V_1, a voltage-gated Na_V_ channel with a cysteine residue in its inactivation domain, responds to local RS by diminished fast inactivation (Fig. 1a) with a sensitivity similar to that of roGFP2^14^. Encouraged by the usefulness of roNa_V_1, we hypothesized that the sensitivity of that sensor might be increased with more reactive selenocysteine (Sec, U) at position 1305. Selenocysteine, the 21st natural amino acid, differs from cysteine by the single substitution of selenium for sulfur. Selenium is a better nucleophile than sulfur and, under physiological conditions, selenocysteine residues are ionized whereas cysteine residues are typically protonated^16^,^17^. Moreover, among functionally characterized mammalian selenoproteins, such as glutathione peroxidases and thioredoxin reductases, selenocysteine is involved in redox reactions catalyzed by these enzymes^18^. These properties render selenocysteine a promising candidate for an RS sensor component in proteins. We hence constructed an expression vector based on rat skeletal muscle Na_V_1.4 channels with a TGA stop codon at the position equivalent to M1305, and followed by a selenocysteine insertion sequence (SECIS) element downstream of the channel gene (Supplementary Fig. 1).

After transient expression of mutant M1305U (roNa_V_2) in HEK 293 cells, currents were measured in the whole-cell mode of the patch-clamp technique. The peak amplitude at −20 mV for roNa_V_2 was much smaller (−0.2 ± 0.02 nA, *n = *20) than for roNa_V_1 (−4.5 ± 0.5 nA, *n* = 20) and wild-type rat Na_V_1.4 (−4.0 ± 0.5 nA, *n* = 20). Supplementation of the cell culture medium with sodium selenate (Na_2_SeO_4_; 300 nM), however, increased the functional expression of roNa_V_2 about 8-fold (−1.4 ± 0.1 nA, *n* = 29; *P* < 0.001), while the mean current amplitudes of roNa_V_1 (−4.3 ± 0.5 nA, *n* = 20; *P* = 0.69) and Na_V_1.4 (−3.6 ± 0.6 nA, *n* = 20; *P* = 0.28) were unaffected (Supplementary Fig. 2).

Kinetics and half-maximal voltage of roNa_V_2 activation were similar to those of Na_V_1.4 or roNa_V_1, whereas the apparent voltage dependence was less steep (Supplementary Fig. 3). The channel inactivation time course at −20 mV was about 4.2-fold slower than for Na_V_1.4 and about 2.4-fold slower than for roNa_V_1 (Fig. 1a,b; Supplementary Fig. 4b).

Production of recombinant selenoproteins requires a complex molecular machinery, which is not completely understood at molecular detail. However, selenium supplement and co-expression of certain trans-acting factors, such as SelA, SelB, and SelC, favors the production of selenoproteins in cultured mammalian cells^19^,^20^. The type of SECIS element and its placement after the stop codon are two additional deciding factors^20^,^21^. Applied to voltage-gated Na^+^ channels in HEK 293 cells, even in absence of the mentioned trans-acting factors, a SECIS element from human selenoprotein N (Supplementary Fig. 1) and supplementation of the cell culture medium with 300 nM sodium selenate as a selenium donor was sufficient to yield measurable Na^+^ current in the range of 100 pA/pF (Supplementary Fig. 2). roNa_V_2 is therefore the first functional Sec-containing ion channel reported and demonstrates the use of Sec insertion as a powerful tool for increasing the experimental realm when studying structure-function relationships.

### Response of roNa_V_2 to endogenous redox stimuli

The presence of an RS-sensitive amino-acid residue, located in the inactivation motif of the channel, provides redox sensitivity to the channel^14^,^22^. Notably, the variability of the non-inactivating current component was larger for roNa_V_2 compared to the other variants (Fig. 1a,b), indicating that the cells are heterogeneous with respect to the basal oxidation level. Furthermore, the fraction of non-inactivating current was diminished when a reducing agent was supplemented to the patch pipette (Supplementary Fig. 4b). Voltage dependence of inactivation and recovery from inactivation showed two components (Supplementary Fig. 4c–f), indicative of part of the channels being already modified at the reactive selenocysteine. However, from a holding potential of −120 mV roNa_V_2 yielded voltage-gated currents that could be stimulated with repetition intervals shorter than 100 ms, thus allowing for a rapid readout.

The relatively large variability of inactivation observed in control HEK 293 cells (Fig. 1a,b, Supplementary Fig. 4b) indicated that roNa_V_2 might be sensitive to the variable ambient intracellular milieu under control conditions. We therefore measured roNa_V_2 currents in HEK 293 cells cultured in media with low (5.5 mM) or high concentrations (25 mM) of glucose as to affect endogenous RS production. The inactivation time course of roNa_V_2 was noticeably slower in the cells cultured in high glucose, while no difference was detected for roNa_V_1 (Fig. 1c,d). On the average, the relative non-inactivating current fraction (R_I_) was about 2.5-fold larger in high glucose compared to low glucose. Furthermore, treatment of the cells with the mitochondrial uncoupler BAM15 (5 μM), which does not depolarize the plasma membrane^23^, reversed this increase to low-glucose levels (Fig. 1d). This indicates that mitochondrial RS, produced as a result of elevated glucose metabolism, can be sensed by membrane proteins, thus defining an effective diffusional space of such RS. Variation of the glucose level did not alter the inactivation properties of roNa_V_1 (Fig. 1e) or the ratiometric fluorescence signal of roGFP2 (Fig. 1f), while for the GSH-dependent cytosolic redox sensor Grx1-roGFP2^6^ similar results as with roNa_V_2 were obtained (Fig. 1g). The experiments also show that under low glucose conditions roNa_V_2 reports reduced conditions of the cytosol being a clear indication that the sensor itself does not significantly disturb the redox milieu as might be expected if the exogenously expressed Na_V_ channels produce some long-lasting Na^+^ influx, thus leading to endogenous RS production as suggested for Na^+^ overload in cardiac myocytes^24^.

Because the current measured in patch-clamp experiments only arises from ion channels in the plasma membrane, the signal provided by roNa_V_2 safely reports membrane-delimited processes, whereas the spatial resolution of fluorescent proteins, even when targeted to the cell membrane, is diffraction limited and confounded by background fluorescence arising from proteins close but not inside the plasma membrane. Moreover, measurements in the cell-attached mode will give access to the status of the sensors positioned under the pipette tip of about 1 μm diameter.

### Response of roNa_V_2 to exogenous RS stimuli

**Figure 2:**
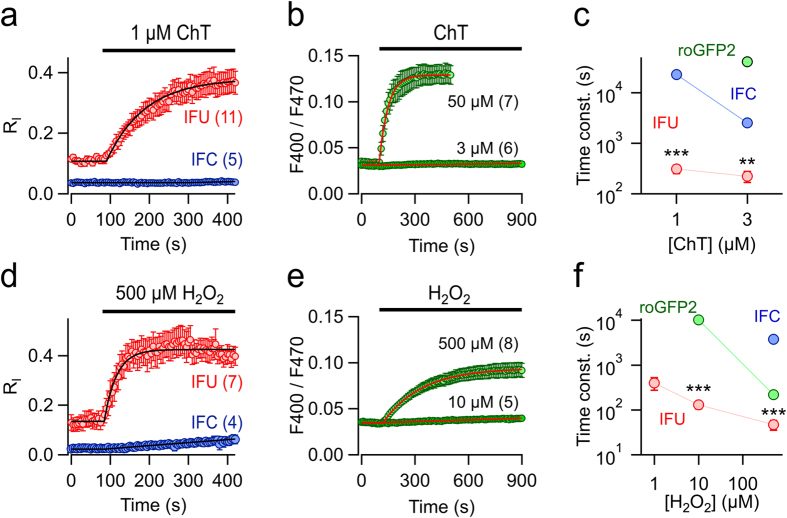
Sensitivity for external stressors. (**a**) Mean time courses of non-inactivating current fraction for the indicated constructs with the application of 1 μM chloramine T (ChT). (**b**) Time courses of the F400/F470 fluorescence ratios of roGFP2 expressed in HEK 293 cells with extracellular application of ChT. (**c**) Time constants of ChT-induced loss of inactivation. The green circle indicates that 3 μM ChT had a negligible effect on the ratiometric roGFP2 signal. (**d**) Mean time courses of non-inactivating current fraction for the indicated constructs with the application of 500 μM H_2_O_2_ (at 32 °C). (**e**) Fluorescence ratios of roGFP2 as in (**b)** for the indicated H_2_O_2_ applications. (**f**) Time constants of H_2_O_2_-induced loss of inactivation (IFC, IFU) and changes in fluorescence ratio (roGFP2). Data are mean ± s.e.m. with *n* indicated in parentheses. ***P < 0.001; **P < 0.01 for a two-sided *t*-test; in (**c)** IFU vs. IFC; in (**f**) IFU vs. roGFP2.

To exclude potential side effects caused by intracellular reactions and to quantify the sensitivity of roNa_V_2 toward chemical modification, HEK 293 cells in the whole-cell configuration were also exposed to extracellular oxidizing agents, and the time course of inactivation loss (R_I_) was described with single-exponential functions. While the oxidant chloramine T (ChT; 3 μM) had a negligible effect on roNa_V_1 within 10 min, roNa_V_2 responded rapidly and robustly (Fig. 2a); the resulting time constants of R_I_ were about 100-fold smaller. Modification of roGFP2 was even slower (Fig. 2b,c). Likewise, application of 500 μM H_2_O_2_ altered inactivation of roNa_V_1 with an extrapolated time constant of 3750 ± 91 s, while roNa_V_2 responded almost 100-times faster (46.6 ± 11.4 s); roGFP2 was intermediate (218 ± 2 s). Even 1 μM H_2_O_2_ application could be readily detected, whereas the response of roGFP2 was about 100-fold slower (Fig. 2d–f).

The data shown thus far clearly demonstrate that roNa_V_2 with a selenocysteine in its inactivation motif reacts about 100-fold faster with RS than roNa_V_1 with a cysteine. A higher sensitivity of a Sec-based compared to a Cys-based RS sensor is anticipated by the strong nucleophilicity of selenocysteine. At physiological pH, selenocysteine is probably deprotonated and more reactive; the p*K*_a_ of selenol/selenate of free selenocysteine is about 5.2, the p*K*_a_ of the thiol/thiolate in free cysteine, however, is 8.3^16^,^17^. Therefore, roNa_V_2 exhibits higher sensitivity to oxidative modification in comparison to roNa_V_1 and roGFP2. Although quantum chemical calculations suggests that the reactivity of Cys and Sel to H_2_O_2_ in aqueous solution is similar, the reactivity of free Sec in aqueous solution is small compared to its reactivity when located in enzymes, for instance in glutathione peroxidase^25^. This indicates that the molecular environment may strongly determine the sensitivity of Sec, and that appears also to be true at the location of the inactivation motif in roNa_V_2 channels.

A rapid response of roNa_V_2 to oxidative modification is critically important for monitoring the occurrence of short-lived RS at small concentrations, particularly when studying membrane-associated processes. Unlike roGFP2 and its variants, engineered Na_V_ channels report on modification(s) of a single amino-acid residue, either Cys (roNa_V_1) or Sec (roNa_V_2), located in the inactivation motif of the channel, i.e. a site right at the interface of membrane protein and cytosol. In particular roNa_V_2 may thus serve as a physiologically important and convenient tool for investigating real-time oxidative cellular events as exemplified for the response to mitochondria uncoupling (Fig. 1d).

### Sensitivity to blue light

**Figure 3:**
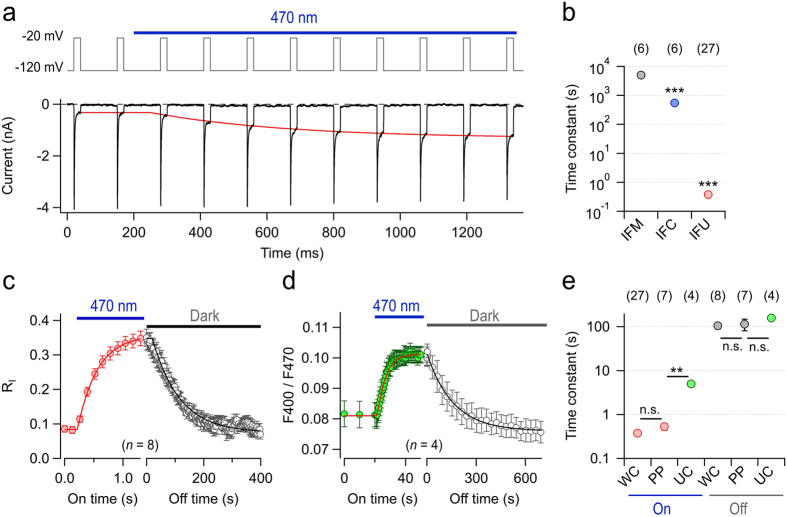
roNa_V_2 senses light-induced chemical modification. (**a**) Current recordings (*bottom*) from a HEK 293 cell expressing roNa_V_2 in response to a train of depolarizing pulses to −20 mV from a holding potential of −120 mV (*top*). The blue bar marks illumination of the cell with 470-nm light via a 63x oil immersion objective. The red curve superimposed to the current traces illustrates a data fit of the time course of light-induced loss of channel inactivation, i.e. increase in steady-state current. Note that the start of the fit is delayed with respect to the light signal because of pertinent channel inactivation (see below). (**b**) Time constants of 470-nm light-induced loss of inactivation for the indicated channel types. (**c**) Mean time course of light-induced loss of inactivation (increase in R_I_) of roNa_V_2 (red) and subsequent recovery in the dark (gray) with superimposed single-exponential fits. (**d**) Mean fluorescence ratio of HEK 293 cells expressing eGFP:147U-204C, i.e. a variant of roGFP2 where one of the disulfide bridge partners is a selenocysteine. The blue bar indicates continuous illumination with 470 nm, the gray bar marks recovery in the dark. (**e**) Mean time constants of light-induced loss of inactivation of roNa_V_2 (“On”) and recovery in the dark (“Off”) comparing experiments performed in whole-cell mode (“WC”) and perforated patch mode (“PP”). Also shown are time constants of fluorescence ratio changes of eGFP:147U-204C (“UC”). Data in (**b–e**) are mean ± s.e.m. with *n* indicated in parentheses. ***P < 0.001; **P < 0.01; n.s., *P* > 0.05 for a two-sided *t*-test; in (**b**) IFU and IFC vs. IFM.

A serious confounding factor in live-cell imaging is that light itself generates RS and initiates photochemical reactions^9^,^10^,^11^,^12^,^13^,^26^, and therefore light-dependent reporters of reactive species have inevitable intrinsic limitations. As we have shown previously^14^, roNa_V_1 inactivation significantly changes when cells under consideration are illuminated with blue light, as usually done when taking GFP fluorescence images. Using the high sensitivity of roNa_V_2, we measured the time course of light-induced chemical modifications at repetition intervals down to 100 ms. Illumination of the cell with blue light (470 nm, 0.5 mW at the microscope objective) resulted in progressive loss of inactivation, characterized by a mean time constant of 0.377 ± 0.042 s (*n* = 27), compared to 549 ± 112 s for roNa_V_1 and 5010 ± 610 s for the wild-type Na_V_1.4 channel (Fig. 3a,b). This rapid change in inactivation properties might be associated with a lasting chemical modification or a short-lived photonic alteration of the selenocysteine residues as reported for certain proteins that contain a diselenide bond^27^. Therefore, we measured reversibility by repetitive current measurements in the dark after a 1-s light exposure and found that inactivation properties were fully restored with a time constant of about 100 s (Fig. 3c). The onset of the sensor response and the time course of reversibility was indistinguishable between recordings performed in the whole-cell configuration, in which the pipette solution has direct contact to the cytosol, and the perforated-patch configuration, where electrical access to the cell interior is mediated by a perforating agent (escin), thus retaining most of the cytosolic components of the cell (Fig. 3e). A higher sensitivity of roNa_V_2 to blue-light-induced RS and rapid recovery in the dark compared to roNa_V_1 is anticipated by a high nucleophilicity and electrophilicity of selenium in Sec compared to sulfur in Cys^28^.

In order to compare roNa_V_2 with fluorescent RS reporter proteins, we also subjected various GFP-based proteins to 470-nm light and measured the change in F400/F470 (Supplementary Fig. 5). roGFP2 responded about as fast as roNa_V_1 (561 ± 37 s, *n* = 4) and GFP mutant S147C (i.e. lacking the partner for forming a disulfide bridge with C204 as present in roGFP2) was even slower (877 ± 18 s, *n* = 5). Even insertion of a selenocysteine at position 147 (eGFP:147U) showed a very weak and slow response (830 ± 14 s, *n* = 5), and in all three cases no reversibility was obtained. Only eGFP mutant 147U-204C showed a fast reaction on blue-light illumination (5.0 ± 0.2 s, *n* = 4), which was fully reversible (158 ± 11 s, *n* = 4) and hence comes close to the performance of roNa_V_2 (Fig. 3d,e). Increase in the speed of S-Se-bond formation as compared to a disulfide bridge formation in roGFP2 may be explained by the longer S-Se bond length of 2.2 Å compared to 2.0 Å for S-S bonds^29^. The similarity of kinetics of the blue-light response observed for eGFP:147U-204C and roNa_V_2 may suggest that the Sec residue in the channel’s inactivation motif may also find a Cys as binding partner to become immobilized. It is also conceivable that light directly influences the formation of a diselenide bond as employed in chemical synthesis^27^.

**Figure 4:**
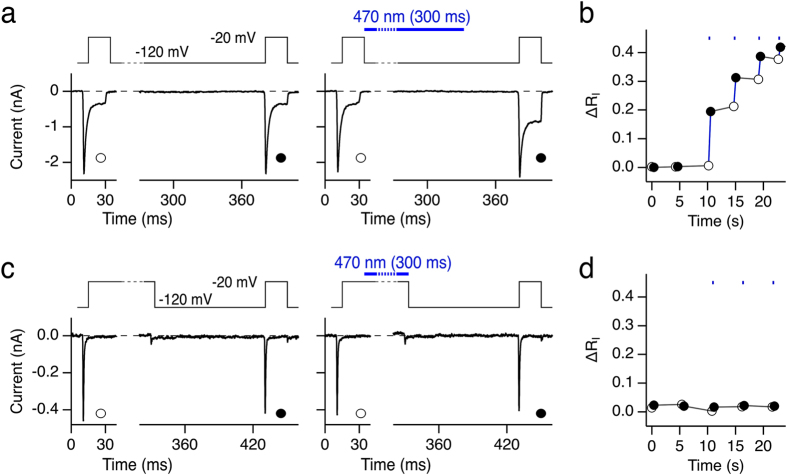
Inactivation protects roNa_V_2 from modification. (**a**) Two successive current traces from HEK 293 cells expressing roNa_V_2 measured with the pulse protocol shown in the top panel. For the first trace no light was on, for the second trace 470-nm light was on between the control and the test pulse as indicated by the blue bar. The interval between the first and the second pulse was about 5 s. (**b**) Relative change in non-inactivating current (ΔR_I_) from the experiment shown in (**a**) and successive sweeps as a function of real-time; white symbols refer to the control pulses, black symbols to the test pulses, blue lines connect consecutive control and test pulses. Episodes of 300-ms blue light exposure are indicated by the blue symbols at the top and manifest in strong increases of R_I_ in the subsequent pulses. (**c**) Similar current recordings as in (**a**) but the blue-light pulse was given while the channel was kept in an inactivated (closed) state by holding the membrane voltage at −20 mV. (**d**) As in (**b**) for the data from (**c**).

The light sensitivity of roNa_V_2 makes it a very useful tool for monitoring light-induced chemical modification at the plasma membrane with a time resolution below 100 ms, and hence it will serve as an important tool for testing for confounding RS production in living cells during photonic experiments. Furthermore, the problem of RS production while using fluorescent proteins for RS detection is avoided. A potential drawback could be the exquisite sensitivity because the sensor itself, just as eGFP mutant 147U-204C (Fig. 3d), is driven into saturation with even short light exposures. However, the sensitivity of roNa_V_2 also depends on the channel state and, hence, the electrical membrane potential. As shown in Fig. 4a, a light pulse applied while the cell was clamped to −120 mV substantially increased R_I_ of roNa_V_2 in the subsequent test pulse. Further brief light pulses result in a cumulative loss of inactivation (Fig. 4b), which follows an exponential function when only the illumination episodes are considered. However, if the light is turned on while the channel is kept in an inactivated state at −20 mV, no modification occurs (Fig. 4c,d) and, thus, the channel sensors are unaffected and ready to be used for further RS sensing.

In summary, roNa_V_2 is not only sensitive and reversible, but also gateable (switchable) by the membrane voltage allowing for complex experimental settings in which membrane-delimited occurrence of reactive species has to be monitored. Complementing other methods of RS determination in living cells^30^, roNa_V_2 adds a non-photonic variant with strict membrane delimitation and demonstrates the power of recombinant selenoproteins as sensitive sensors for redox processes.

## Methods

### Vector construction and mutagenesis

The wild-type Na^+^ channel construct used in this study was based on rat Na_V_1.4 (SCN4A, P15390^31^) in the plasmid vector pcDNA3. Mutants thereof were generated by site-directed mutagenesis and verified by DNA sequencing (also see ref. 22). Point mutants were introduced into the inactivation motif 1303IFM and thus mutants are termed by this motif only, “IFU” for mutant M1305U, and “IFC” for mutant M1305C. A TGA stop codon was introduced between nucleotides 3913 and 3915 of the rNa_V_1.4 sequence using a PCR-based strategy, replacing the original ATG codon. A SECIS element was isolated from the human selenoprotein N (SELENON, NM_206926) downstream sequence and inserted after the original stop codon of the sequence of rNa_V_1.4, as shown in Supplementary Fig. 1a. This construct was inserted in a mammalian pcDNA3 expression vector. roGFP2^5^, Grx1-roGFP2^6^, GFP-variants with selenocysteine^32^, and all further GFP-based mutants, and CD8 were in pcDNA3 plasmids. The reading frame for selenocysteine-containing GFP was followed by the same SECIS element as used in the rNa_V_1.4 construct.

### Cell culture and transfection

HEK 293 cells (CAMR, Porton Down, Salisbury, UK) were maintained in Dulbecco’s Modified Eagle’s Medium (DMEM) mixed 1:1 with Ham’s F12 medium and supplemented with 10% fetal calf serum in a 5% CO_2_ incubator at 37 °C. Cells were trypsinized, diluted with culture medium, and grown in 35-mm dishes. HEK 293 cells were transfected with the respective plasmids using the Roti^®^-Fect transfection reagent (Roth, Karlsruhe, Germany) following the instructions of the supplier. Grown cells were supplemented with sodium selenate (Na_2_SeO_4_; 300 nM) for 12 hours from the day of transfection. After 12 hours, transfected cells were maintained in fresh cell culture media without sodium selenate. Electrophysiological experiments were performed 1–5 days after plating. Cells not expressing a fluorescence protein were cotransfected with CD8 to identify transfected cells by means of anti-CD8-coated beads (Deutsche Dynal GmbH, Hamburg, Germany).

### Electrophysiological recordings

Whole-cell voltage-clamp experiments were performed as described previously^33^. Briefly, patch pipettes with resistances of 0.7–1.5 MΩ were used. The series resistance was compensated for by more than 70% in order to minimize voltage errors. Perforated-patch recordings were performed by adding escin at 1–10 μM^34^ to the patch pipette solution yielding a series resistance between 3 and 20 MΩ after about 15 minutes in the on-cell configuration.

A patch-clamp amplifier EPC10 was operated by PatchMaster software (both HEKA Elektronik, Lambrecht, Germany). Holding potential was −120 mV. Leak and capacitive currents were corrected with a *p*/4 method with a leak holding voltage of −120 mV. Currents were low-pass filtered at 5 kHz and sampled at a rate of 25 kHz. Most experiments were performed at constant temperature of 19–21 °C; 32 °C were chosen for the assessment of H_2_O_2_ effects on HEK cells.

The patch pipettes contained (mM): 35 NaCl, 105 CsF, 10 EGTA, 10 HEPES (pH 7.4 with CsOH). The bath solution contained (mM): 150 NaCl, 2 KCl, 1.5 CaCl_2_, 1 MgCl_2_, 10 HEPES (pH 7.4 with NaOH).

Channel activation and inactivation was assessed by depolarizing pulses to −60 through 60 mV in steps of 10 mV at an interval of 2 s from a holding potential of −120 mV. The peak current-voltage relationships were fit according to a Hodgkin-Huxley formalism with *m* = 3 activation gates and a single-channel conductance according to the Goldman-Hodgkin-Katz equation.


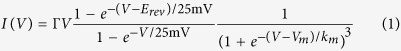


V_m_ is the voltage of half-maximal gate activation and k_m_ the corresponding slope factor. Γ is the maximal conductance of all channels and E_rev_ the reversal potential.

Steady-state inactivation was measured from a holding potential of −120 mV with conditioning pulses of 500 ms at voltages ranging from −120 to −45 mV in steps of 5 mV. Subsequently, peak current was determined at −20 mV and normalized to a control peak current measured before conditioning. The repetition interval was 10 s. The normalized peak current plotted versus the conditioning voltage was described with a two-component Boltzmann function:


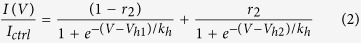


with the half-maximal inactivation voltages, V_h_, and the corresponding slope factors, k_h_, which indicate the voltage dependence of inactivation.

The time course of oxidation-induced loss of inactivation was monitored by measuring the ratio of current at 5 ms (I_5_) or 10 ms (I_10_) after the depolarization onset and the peak current (I_p_) elicited by pulses to −20 mV (R_I_). 5 ms was chosen for cases were a direct comparison with wild-type channels and roNa_V_1 was intended because the inactivation of those channels is faster that that of roNa_V_2. The remaining non-inactivating remaining current fraction, R_I_(t), was plotted as a function of time (t) and fit with the following function:


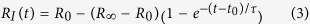


with the ratio before oxidation, R_0_, the time of oxidation start, t_0_, the time constant, τ, and the ratio after infinite exposure, *R*_*∞*_.

Data were analyzed with FitMaster (HEKA Elektronik) and IgorPro (WaveMetrics, Lake Oswego, OR, USA). If not stated otherwise, averaged data are presented as mean ± s.e.m. (*n* = number of independent measurements). Groups of data were compared with a two-sided Student’s *t*-test or ANOVA followed by a *post hoc* Bonferroni correction when appropriate.

Chemicals. Chloramine-T (ChT) and hydrogen peroxide (H_2_O_2_) were diluted in the respective bath solution immediately before application. The oxidant was applied extracellularly with a fine-tipped application pipette. In some experiments the reducing agent TCEP (tris(2-carboxyethyl) phosphine hydrochloride) was added to the internal patch pipette solution at 1 mM concentration with readjustment of the pH. BAM15 was from TimTec (Newark, DE, USA).

### Fluorescence measurements

Fluorescence of individual cells expressing roGFP2 or other GFP-based mutants was measured with a photodiode (TILL Photonics, Gräfelfing, Germany). Excitation light of 400 and 470 nm (width about 15 nm) from a PolyChrome-V light source (TILL Photonics) was applied for 10 ms each. Fluorescence ratio (F400/F470) was formed based on the mean fluorescence values of the trailing 5-ms intervals. Filter set: 492/SP, FT 495, HC 520/35 (AHF Analysentechnik, Tübingen, Germany).

### Photonic stimulation

RS were generated by epifluorescence excitation light. This was achieved with light from the monochromator set to 470 nm, using a 63x/1.4 oil objective and the GFP filter set as mentioned above.

## Additional Information

**How to cite this article**: Ojha, N. K. *et al*. Non-photonic sensing of membrane-delimited reactive species with a Na^+^ channel protein containing selenocysteine. *Sci. Rep.* **7**, 46003; doi: 10.1038/srep46003 (2017).

**Publisher's note:** Springer Nature remains neutral with regard to jurisdictional claims in published maps and institutional affiliations.

## Supplementary Material

Supplementary Information
