# Supplementary material for: Non-photonic sensing of membrane-delimited reactive species with a Na+ channel protein containing selenocysteine
Source: Sci Rep. 2017 Apr 5;7:46003. doi: 10.1038/srep46003 (PMC5381000; doi:10.1038/srep46003)
Supplement: Supplementary Information [file srep46003-s1.pdf]

Non-photonic sensing of membrane-delimited reactive species with a Na^+ channel protein containing selenocysteine

– Supplementary Material –

Navin K. Ojha[1], Enrico Leipold[1], Roland Schönherr[1], Toshinori Hoshi[2], and Stefan H. Heinemann[1]

Corresponding author:

Prof. Dr. Stefan H. Heinemann

Center for Molecular Biomedicine, Department of Biophysics,

Friedrich Schiller University Jena & Jena University Hospital,

Hans-Knöll-Str. 2, D-07745 Jena, Germany

Tel: ++49-3641-9 39 56 50

Fax: ++49-3641-9 39 56 52

E-Mail: Stefan.H.Heinemann@uni-jena.de

Running title: roNa$_V$2

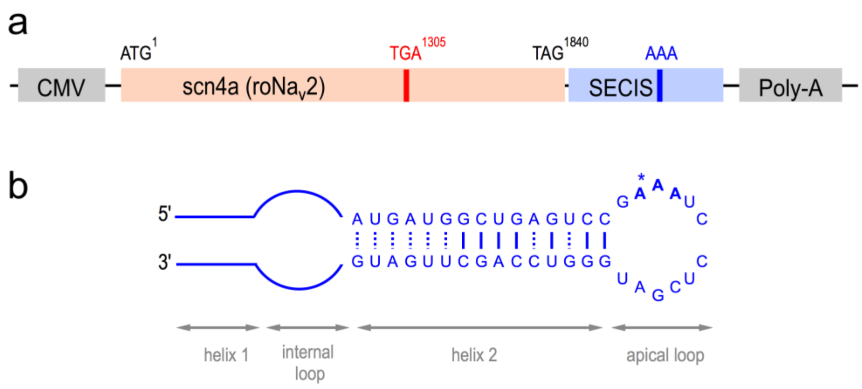

Supplementary Figure 1. Vector construction for selenocysteine incorporation into Na_V1.4. (**a**-**b**) A 225-bp fragment containing nucleotides 2811-3065 from human SELENON mRNA (NM_020451.2, SEPN1) was placed directly after the endogenous scn4a stop codon TAG, thereby placing the AAA consensus sequence of the SECIS element (**b**) 140 nt downstream of the scn4a stop codon.

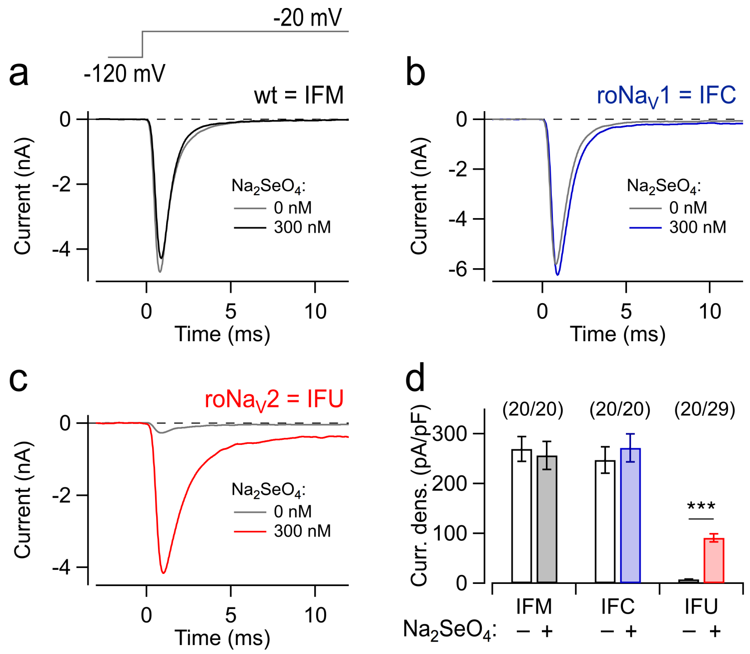

Supplementary Figure 2. Influence of selenate on the functional expression level. (**a**-**c**) Representative current traces of the indicated channel constructs, expressed in HEK 293 cells, either in normal medium (0 nM Na_2SeO_4.) or in medium supplemented with 300 nM Na_2SeO_4. (**d**) Mean current density at –20 mV of the indicated channel constructs cultured in medium without (white bars) or medium with 300 nM Na_2SeO_4 (colored bars). Data in d are mean ± s.e.m. with *n* indicated in parentheses; ***, $P < 0.001$ for a *t*-test.

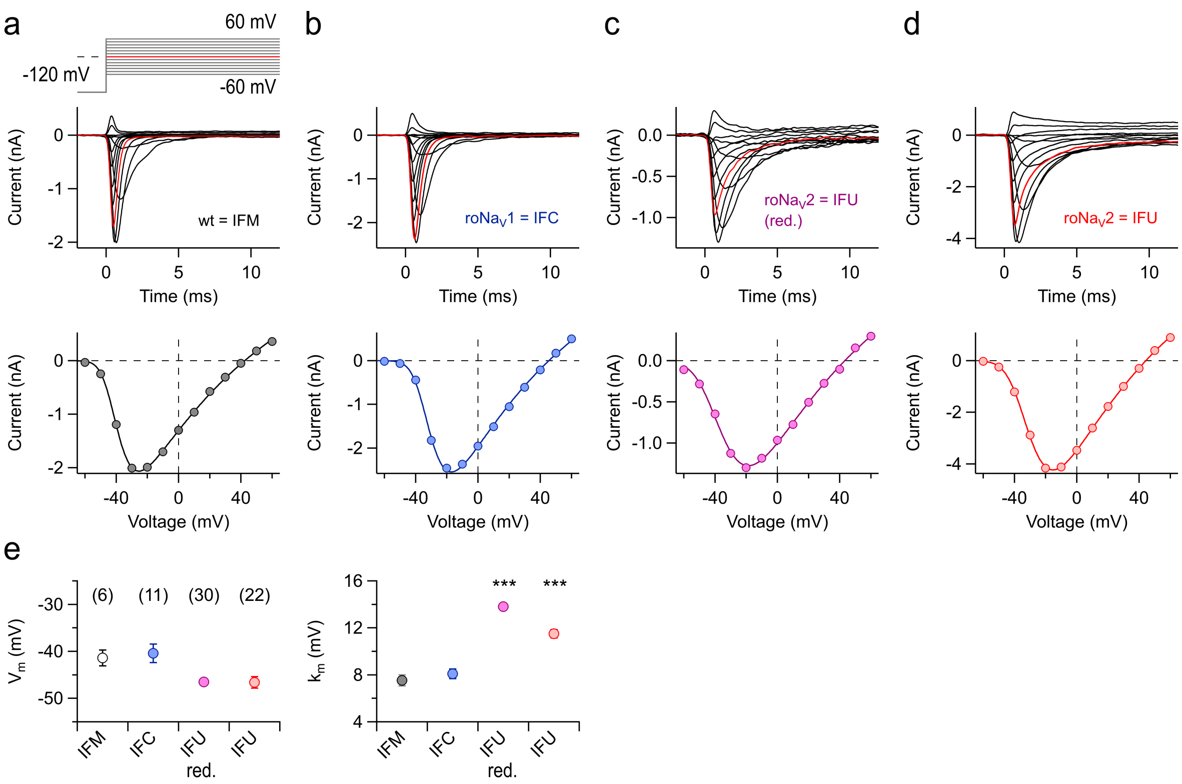

Supplementary Figure 3. Voltage-dependent activation of Na$_V$1.4 channels with variations at position 1305. (**a**) Top: Superposition of current traces of Na$_V$1.4 channels expressed in HEK 293 cells in response to the indicated pulse paradigm. The current trace at 0 mV is shown in red. Bottom: Peak currents as a function of voltage with superimposed fit according to Eq. 1. (**b**) As in a, but for mutant Na$_V$1.4-M1305C (also termed roNa$_V$1 or IFC). (**c**) As in a, but for mutant Na$_V$1.4-M1305U (also termed roNa$_V$2 or IFU) using a pipette solution supplemented with 1 mM TCEP. (**d**) As in c, but without reducing agent. (**e**) Results of the current-voltage fits: half-maximal voltage of the m-gate activation, V_m, (left) and the corresponding voltage dependency, k_m, (right). Data are mean ± s.e.m. with *n* indicated in parentheses. *** $P < 0.001$ for a *t*-test versus wild type (IFM) after the Bonferroni correction for multiple comparisons.

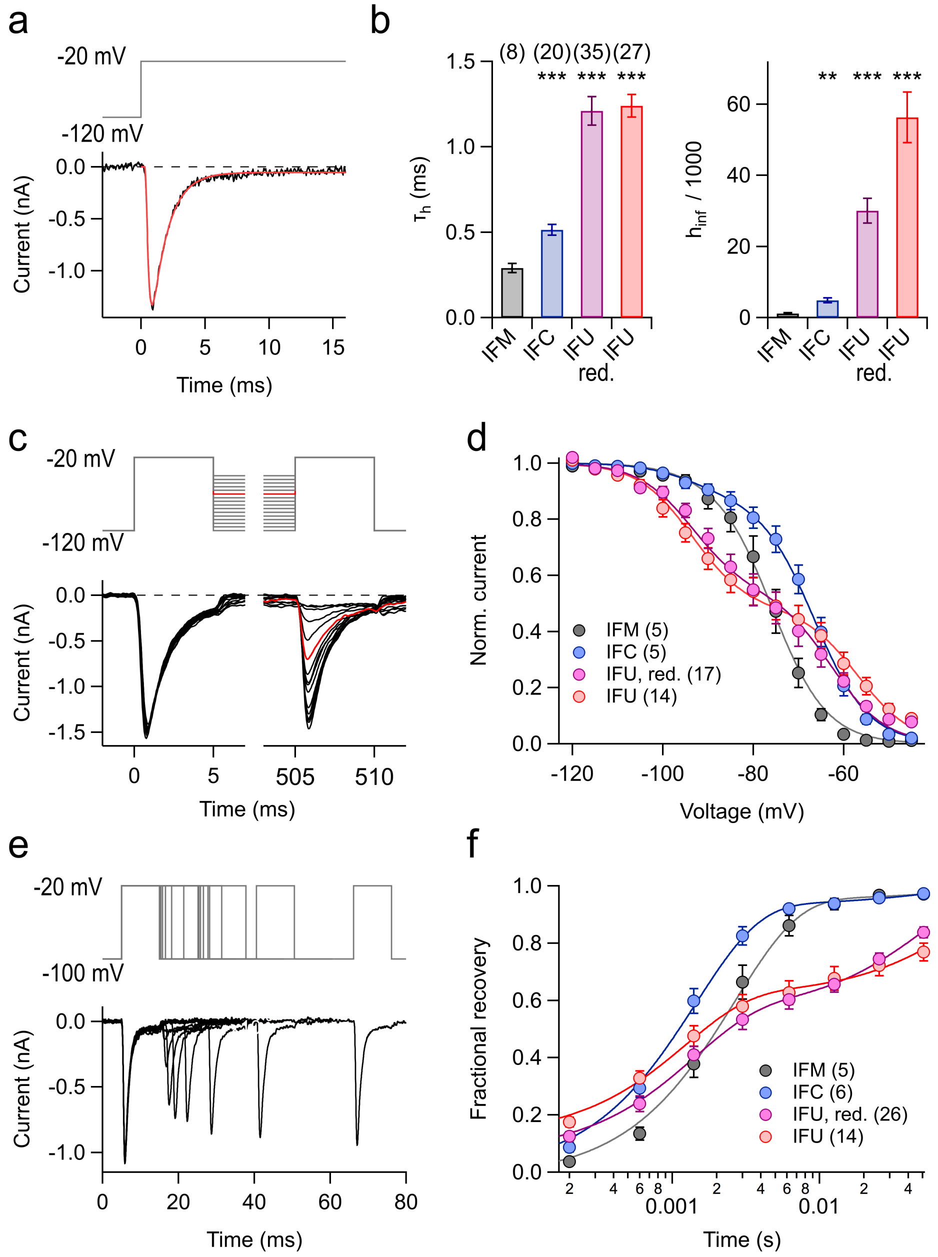

Supplementary Figure 4. Inactivation properties. (**a**) Current trace of Na$_V$1.4-M1305U (IFU; black) for the indicated pulse protocol with superimposed fit (red) according a Hodgkin-Huxley formalism with 3 activation and one inactivation gates yielding the time constant of inactivation, τ_h, and an estimate for the steady-state inactivation, h_∞. (**b**) Analysis results from fits as shown in (a) characterizing the time constant of inactivation (τ_h, left) and the steady-state inactivation (h_∞, right) for the indicated amino acids at position 1305. (**c**) Pulse protocol and superimposed sample data for the assessment of

the voltage dependence of inactivation; the interpulse intervals lasted 500 ms. The trace at –70 mV is shown in red. (**d**) Peak current measured during the second depolarization relative to the control pulse as a function of conditioning voltage with superimposed global fits according to Eq. (2). V_{h1} was constrained for all variants: –93.5 ± 0.5 mV, the slope factors k_h were constrained for both components and all variants: 5.98 ± 0.20 mV. V_{h2} and the fractional contribution of that component were fit individually: IFM, –75.7 ± 0.6 mV, 5.0 ± 3.4%; IFC, –66.5 ± 0.4 mV, 11.7 ± 1.7%; IFU (red.), –62.7 ± 0.7 mV, 45.6 ± 1.6%; IFU, –56.8 ± 0.7 mV, 51.8 ± 1.3%. (**e**) Pulse protocol and sample current traces for the assessment of the time course of recovery from inactivation. (**f**) Fractional recovery at –100 mV as a function of the interpulse interval with superimposed double-exponential fits yielding two time constants and the fractional contribution of the fast time component: IFM, 2.65 ± 0.27 ms, 96 ± 283 ms, 94.2 ± 4.1%; IFC, 1.45 ± 0.12 ms, 59 ± 66 ms, 93.2 ± 2.7%; IFU (red.), 1.34 ± 0.95 ms, 50.0 ± 3.8 ms, 52.9 ± 1.4%; IFU, 1.21 ± 0.13 ms, 92.8 ± 16.6 ms, 56.4 ± 1.9%. Data are mean ± s.e.m. with *n* indicated in parentheses. ** $P < 0.01$, *** $P < 0.001$ for a *t*-test versus wild type (IFM) after the Bonferroni correction for multiple comparisons.

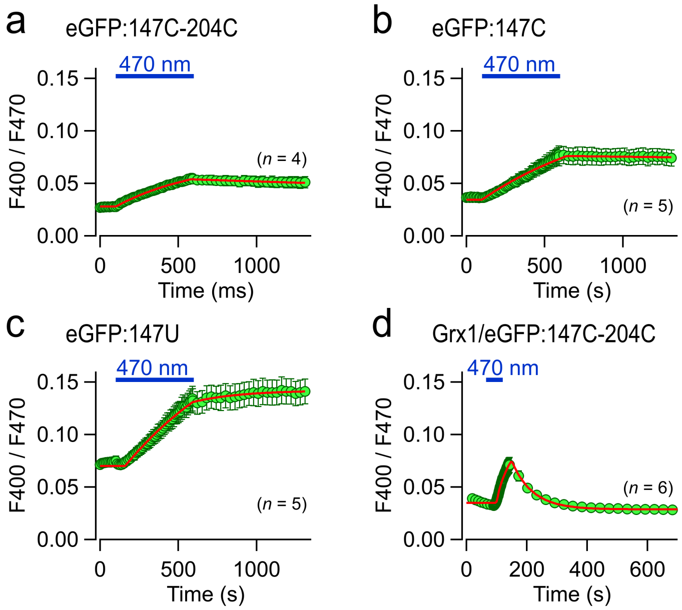

Supplementary Figure 5. Blue-light dependence of eGFP-based fluorescence proteins. Time courses of the F400 / F470 fluorescence ratios of the indicated eGFP-based proteins expressed in HEK 293 cells with blue-light stimulation via a 63x objective indicated by the blue bars. Data are mean ± s.e.m. with *n* indicated. (**a**) roGFP2 (i.e. eGFP:S147C-Q204C), (**b**) eGFP:S147C, (**c**) eGFP:S147U, and (**d**) Grx1-roGFP2 (i.e. Grx1 fused to eGFP:S147C-Q204C). The superimposed red curves are the results of single-exponential fits during and after light exposure.
